# Drug resistance in glioblastoma: from chemo- to immunotherapy

**DOI:** 10.20517/cdr.2023.82

**Published:** 2023-10-11

**Authors:** Sachin Sharma, Oksana Chepurna, Tao Sun

**Affiliations:** Department of Neurosurgery, Cedars-Sinai Medical Center, Los Angeles, CA 90048, USA.

**Keywords:** Glioblastoma, immunotherapy, drug resistance, tumor microenvironment, immunosuppression

## Abstract

As the most common and aggressive type of primary brain tumor in adults, glioblastoma is estimated to end over 10,000 lives each year in the United States alone. Stand treatment for glioblastoma, including surgery followed by radiotherapy and chemotherapy (i.e., Temozolomide), has been largely unchanged since early 2000. Cancer immunotherapy has significantly shifted the paradigm of cancer management in the past decade with various degrees of success in treating many hematopoietic cancers and some solid tumors, such as melanoma and non-small cell lung cancer (NSCLC). However, little progress has been made in the field of neuro-oncology, especially in the application of immunotherapy to glioblastoma treatment. In this review, we attempted to summarize the common drug resistance mechanisms in glioblastoma from Temozolomide to immunotherapy. Our intent is not to repeat the well-known difficulty in the area of neuro-oncology, such as the blood-brain barrier, but to provide some fresh insights into the molecular mechanisms responsible for resistance by summarizing some of the most recent literature. Through this review, we also hope to share some new ideas for improving the immunotherapy outcome of glioblastoma treatment.

## INTRODUCTION

Brain tumors affect more than ~17,000 people in the United States each year, where gliomas are considered the most common type of primary brain tumor^[1]^. Glioblastoma is a grade IV astrocytoma that was initially categorized into four molecular subtypes, termed neural, proneural, classical, and mesenchymal subtype^[2]^. Transcriptional profiling and genetic modeling in mice showed that glioblastoma originated from neural stem cells (NSC), NSC-derived astrocytes, and oligodendrocyte precursor cells (OPCs)^[3-5]^. Besides the four molecular subtypes based on their transcription profiling, glioblastoma tumors can also be classified by the status of the isocitrate dehydrogenase gene (IDH) as IDH wild-type and IDH-mutant tumors. Similarly, epigenetics factors, such as CpG island methylation phenotype of O6-methylguanine-DNA methyltransferase (MGMT) promoter, are also commonly used for glioblastoma tumor stratification^[6,7]^.

Since the approval of Temozolomide (TMZ) for newly diagnosed glioblastoma treatment by the FDA in early 2000, surgery followed by radiotherapy and TMZ treatment has remained the first-line glioblastoma treatment^[8]^. However, none of these therapies eliminate cancer cells entirely because of challenges marred by high infiltration rate, tumor heterogeneity, blood-brain barrier (BBB), and immunosuppressive environment factors^[9,10]^. The highly infiltrative nature of glioblastoma does not allow the removal of cancerous cells using resection; self-renewing cells followed by resection become more prone to radioresistance and chemoresistance. Similarly, cellular heterogeneity and BBB prevent targeted drug delivery in glioblastoma^[11,12]^.

## COMMON DRUG RESISTANCE MECHANISMS IN BRAIN CANCERS

### Blood-brain barrier

Although the BBB in glioblastoma is compromised to some extent, tumor BBB still presents a great challenge for therapeutics to reach glioblastoma cells. As the intrinsic barrier for brain cancer, BBB is a microvasculature structure surrounding the central nervous system (CNS), tightly regulating the movement of molecules and cells between the CNS and blood. Normally, BBB maintains the homeostasis of CNS and prevents infiltration of toxins, pathogens, inflammation, and harmful metabolites^[13-15]^. Disruption of the neurovascular unit (NVU) is associated with blood-brain dysfunction in neurodegenerative disease and brain tumors^[16]^. The NVU consists of vascular cells (endothelial, pericytes, and vascular smooth muscle cells), glia (astrocytes, microglia, and oligodendroglia), and neurons, and it plays an important role in maintaining BBB functional integrity and regulating the volume of cerebral blood flow^[17,18]^. The endothelial cells in neurovascular parenchyma form capillary beds connected through tight junctions (TJs), surrounded by a specialized basal lamina shared with pericytes and astrocytic end feet. They are sparsely interconnected by neuronal endings and microglia^[19,20]^. Astrocytes and pericytes, an essential constituent of NVU, release Sonic Hedgehog and vitronectin and angiopoietin I, respectively, acting on endothelial cells for their survival and maintaining BBB.

### Overexpression of efflux pumps

Efflux transporters on the BBB membrane also contribute to cerebrospinal fluid homeostasis by protecting it from potentially harmful endogenous and exogenous substances^[21,22]^. These transporters also pose challenges by blocking therapeutic compounds from entering the brain parenchyma. Efflux transporters on compartments of the BBB belong to either ATP-binding cassette (ABC) or the solute carrier (SLC) superfamilies^[23,24]^. Organic anion-transporting polypeptides (OATP) are a superfamily of solute carrier organic anionic (SLCO) transmembrane transporters that are known for cancer drug resistance^[25,26]^. These peptide transporters regulate a variety of xenobiotic and endogenous substrates, including endogenous hormones, their conjugates, and anticancer drugs^[27]^. OATP1A2 is a sodium-independent uptake transporter family member and is highly expressed on the luminal membrane of BBB in tumors and adjacent healthy tissues^[28]^. A study by Cooper *et al.* in glioblastoma patients showed significant over-expression of all the OATP isoforms (OATP1A2, 2B1, 1C1, and 4A1) in tumor tissues compared to non-neoplastic brain^[29]^.

### Enhanced DNA damage repair pathways (MGMT) and abnormal activation of survival signaling pathways

As part of the glioblastoma standard treatment regimen, TMZ is a potent DNA alkylating agent that leads to DNA damage in cancer cells and cell death^[30]^. However, TMZ treatment often results in drug resistance in ~50% of glioblastoma patients due to overexpression of MGMT, which reverses the methylation of the O6 position of guanine. In addition to upregulated MGMT expression, glioblastoma often exhibits enhanced DNA damage repair capacity through several related mechanisms. For instance, poly(ADP-ribose) polymerase (PARP) was shown to interact with MGMT and enhance MGMT function in the removal of O6-methylation of DNA^[31]^. Interestingly, even in MGMT-deficient glioblastoma, TMZ resistance may still arise due to the loss of mismatch repair (MMR) pathway in tumor cells. Recent work by Lin *et al.* developed a new class of compound (KL-50) to achieve MMR-independent glioblastoma cell killing. It demonstrated a promising strategy to exploit cancer-specific deficiencies in DNA repair pathways^[32]^. Glioblastoma tumors also have elevated levels of receptor tyrosine kinases, such as EGFR gene amplification or mutation (EGFRvIII), PDGFR and FGFR, and aberrant activation of PI3K/ATK signaling and other growth factors (e.g., IGF-1, CTGF, and TGFβ)^[33-39]^, with a potential contribution to the drug resistance phenotype.

### Role of glioma stem cells

Glioma stem cells (GSCs) represent a subpopulation of relatively undifferentiated cells capable of self-renewal while also generating clonal populations of differentiated tumor cells in glioblastoma. These cells are increasingly recognized as a driving force supporting glioma genesis, therapy resistance, and recurrence^[40]^. GSCs have high regenerative capacity and can differentiate into cells expressing several lineage markers such as CD133, SOX2, CD15, CD44, integrin α6, and CD36^[41]^. Along with heterogeneity, various factors contribute to the chemoresistance of GSCs. Intrinsic factors include upregulated MGMT, higher anabolic capacity, and autophagy-mediated clearance of ROS induced by chemotherapy. Extrinsic factor is mainly hypoxic tumor microenvironment (TME). Hypoxia promotes the expression of GSC markers and a cancer stem-like phenotype^[42]^. Hypoxia-response genes, such as hypoxia-inducible factor HIF-2α and VEGF, are highly expressed in GSCs. Intriguingly, two reports have demonstrated that hypoxia-associated transcriptional signatures can be used as prognostic markers for glioblastoma patients^[43,44]^.

### Epigenetic modulations

Epigenetic dysregulation has been increasingly recognized as one of the significant drivers of oncogenesis, and several subtypes of glioblastoma are associated with epigenetic alterations^[45,46]^. These epigenetic modifications may serve as valuable biomarkers for tumor stratification and prognostic prediction. For instance, the glioblastoma resistance to receptor tyrosine kinase (RTK) inhibitors has been found to involve both genetic and epigenetic mechanisms^[47]^, resulting in subclones with a gain of copy number in the insulin receptor substrate-1(IRS1) and substrate-2 (IRS2) loci. Another study identified a long non-coding RNA (LINC00021) that promotes TMZ resistance through Notch signaling and epigenetically silenced p21 expression via recruiting EZH2^[48]^, one of the methyltransferases responsible for histone methylation. Epigenetic modifications in glioblastoma are also exploited as drug targets. Among the promising epigenetic interventions for glioblastoma are the histone deacetylase (HDAC) inhibitors^[49]^, which have been extensively tested in various cancers^[50]^. HDAC inhibitors can block cancer cell proliferation by inducing cell cycle arrest, cell differentiation, and/or apoptosis^[51]^. With a large amount of supportive preclinical data, various HDAC inhibitors in glioblastoma clinical trials are underway.

## DRUG RESISTANCE TO IMMUNOTHERAPY IN GLIOBLASTOMA

### Current status of immunotherapy trials in glioblastoma

Although immune checkpoint inhibitors have greatly improved cancer treatment today, the clinical trials in glioblastoma treatment have been largely unsuccessful.

**Figure 1:**
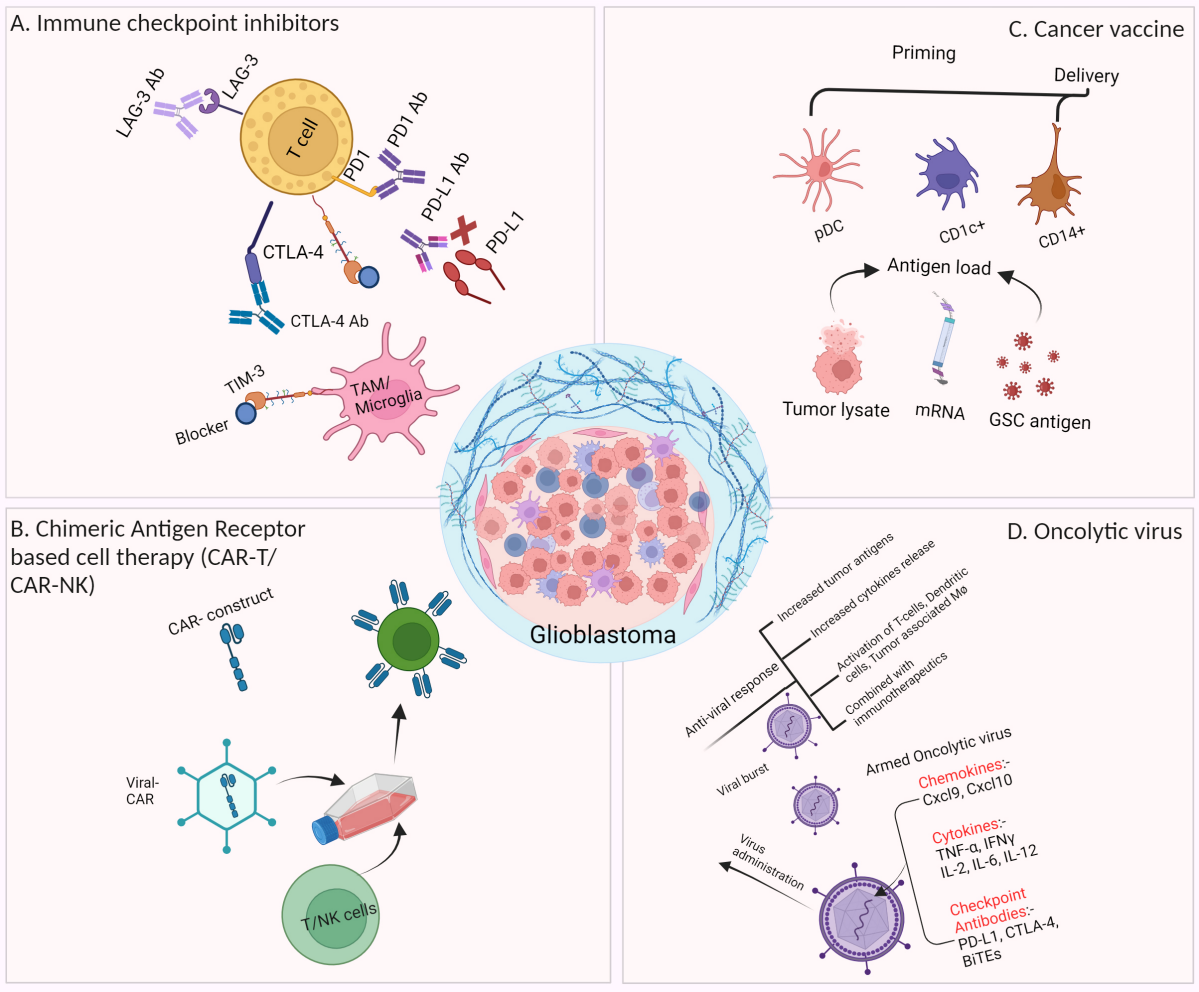
Various forms of immunotherapy in preclinical and clinical trials for glioblastoma treatment. (A) Various checkpoint inhibitors, including α-PD1, α-PD-L1, α-CTLA-4, and α-TIM-3, have been studied in glioblastoma treatment; (B) CAR-based adoptive cell therapies have attained immense success against hematopoietic cancer, but have shown limited effects on glioblastoma; (C) Cancer vaccine has been tested in glioblastoma treatment by priming antigen-presenting cells (e.g., Dendritic Cells) with tumor antigens/lysate or synthetic antigen peptides, followed by infusion back to the patients; (D) An OV can lyse tumor cells through replication. OV can be armed with immunotherapy in which a virus is genetically modified to carry checkpoint inhibitors (e.g., α-PD-L1 and α-CTLA-4), therapeutic proteins, chemokine (Cxcl9, Cxcl10) or cytokines genes (IFNγ, IL-6, IL-12). Those armed OVs are more potent in killing cancer cells^[52]^. (Created with BioRender.com). CTLA-4: Cytotoxic T-lymphocyte-associated protein 4; GSC: glioma stem cell; LAG-3: lymphocyte activation gene-3; OV: oncolytic virus; TAM: tumor-associated macrophage.

We summarized the most common immunotherapies that have been evaluated in glioblastoma in either preclinical or clinical trials [Figure 1]. The most widely tested immunotherapies in glioblastoma (like in all other cancers) are immune checkpoint inhibitors (ICIs). Immune checkpoint molecules are typically expressed on the surface of immune cells, and they play a crucial role in maintaining immune balance, preventing excessive immune activation, and avoiding auto-immune response. This function of immune regulation is achieved through the interaction of immune checkpoints with their corresponding ligands on other cells, and cancer cells often hijack this communication mechanism to suppress the anti-tumor immunity and evade immune surveillance^[53-55]^. A common working mechanism of ICIs is to block the inhibitory signal to the immune cells (usually from cancer cells) through an antibody binding to the checkpoint or its ligand to disengage their interaction. Since the discovery of the first immune checkpoint, cytotoxic T-lymphocyte-associated protein 4 (CTLA-4), more than a dozen of these checkpoint molecules have been identified to date, such as PD1 and its ligand PD-L1/L2, TIM3, lymphocyte activation gene-3 (LAG3), and TIGIT^[54]^. Among various ICIs, α-PD-1 has been widely studied as a monotherapy^[56]^ or in a combination of either radiation or radiation plus TMZ in multiple trials (CheckMate 143, 498 and 548)^[57-59]^. Overall, the clinical outcome has been rather murky in both primary and recurrent glioblastoma due to multiple resistance mechanisms, including high tumor heterogeneity, low mutational burden, systemic immunosuppression, and local immune dysfunction^[60]^.

CAR-T therapy has been studied in glioblastoma^[61]^. The targets of these CARs in clinical trials span from growth signaling receptors (EGFR/EGFRvIII, Her2), cytokine receptors (IL13Rα2), immune checkpoint (B7-H3) to even matrix metalloproteinase (MMP2), and disialoganglioside (GD2)^[62]^. Besides very limited responders, including pediatric patients with diffuse intrinsic pontine glioma (DIPG)^[63,64]^, most trials failed to demonstrate a sustained clinical benefit, mainly due to CAR-T-associated severe side effects, including cytokine release syndrome and high grade of neurotoxicity^[65,66]^.

Cancer vaccines have also been explored in glioblastoma trials with minimal success. A peptide vaccine targeting EGFRvIII called rindopepimut has been tested in various trials, with only one trial (phase II) reporting a marginal increase in median overall survival of 12.0 months with rindopepimut plus bevacizumab compared to 8.8 months with bevacizumab plus vaccine placebo^[67]^. The main limitation of EGFRvIII vaccine is that the expression of EGFRvIII is only limited in some glioblastoma patients, and there is also an intra-tumoral heterogeneous pattern of EGFRvIII expression, which further hinders the overall immune response to the tumor. Another cancer vaccine strategy is to use patient-derived dendritic cells with ex vivo exposure to glioblastoma neoantigens. For instance, ICT-107 and DCVax-L both used patient autologous dendritic cells with pulse to either peptides designed based on patient tumors (ICT-107) or autologous tumor lysates (DCVax-L). Both trials have reached phase 3 and had an acceptable safety profile, though the efficacy was minimal^[68,69]^.

Oncolytic virus (OV) can be viewed as a gene & immuno-hybrid therapy. Typically, an OV exerts its anti-tumor function through a dual mode of action - tumor cell killing (lysis) and induction of systemic anti-tumor immunity^[70]^. An OV can selectively infect and lyse cancer cells, and various viruses have been employed to develop oncolytic viruses^[71]^. Upon lysis of tumor cells due to OV replication, many tumor antigens will be released, leading to a local and systemic anti-tumor reaction^[72]^. One of the main issues associated with OV therapy is the host’s anti-viral immune response to the OV^[73]^. Currently, a modified herpes simplex virus type 1, named teserpaturev or G47Δ, is the only OV that received conditional approval (in Japan) for glioblastoma treatment^[74]^, and many more oncolytic viruses are currently in clinical trials for glioblastoma treatment (reviewed by Suryawanshi & Schulze^[75]^). Among them, a retroviral OV called Toca511 reached phase III clinical trial, but was terminated due to its failure to improve survival and meet other endpoints^[75]^.

### Immunosuppressive TME

Glioblastoma tumors generally have a low to moderate mutation rate, especially compared to other solid tumors such as melanoma, non-small cell lung cancer, GI cancer, and head and neck cancer^[76]^. The tumor mutation burden was found to be correlated with immunotherapy treatment response^[77]^. In addition, glioblastoma also has a highly immune-suppressive microenvironment with a large amount of infiltrating myeloid cells, including bone marrow-derived macrophages (MΦ), myeloid-derived suppressor cells (MDSCs), dendritic cells (DCs), and neutrophils^[78]^. T lymphocyte dysfunction in the glioblastoma is very severe and was found to be mediated partially by IL-10 produced by the myeloid cells^[79]^. Additionally, within the TME, prolonged antigen exposure to T cells leads to the expression of LAG3, which in turn causes T cell exhaustion^[80]^. More strikingly, patients with glioblastoma also have systemic immune suppression. For instance, glioblastoma patients have lower numbers of circulating T cells due to the sequestration of T cells in the bone marrow, possibly due to loss of sphingosine-1-phosphate receptor 1 (S1P1) expression^[81]^. S1P1 is a GPCR that binds the lipid second messenger, sphingosine-1-phosphate (S1P), and the S1P-S1P1 axis plays a pivotal role in lymphocyte trafficking^[82]^. Typically, surface S1P1 affords T cell egress from the spleen, lymph node, and thymus. In a mouse glioblastoma model, the T cells from tumor-bearing mice were found to have lost surface expression of S1P1, leading to T cells sequestered mainly in bone marrow^[81]^. This may partially explain the T cell lymphopenia in glioblastoma patients. However, treatment (radiation and TMZ) associated T cell lymphopenia was also very common^[83,84]^.

**Figure 2:**
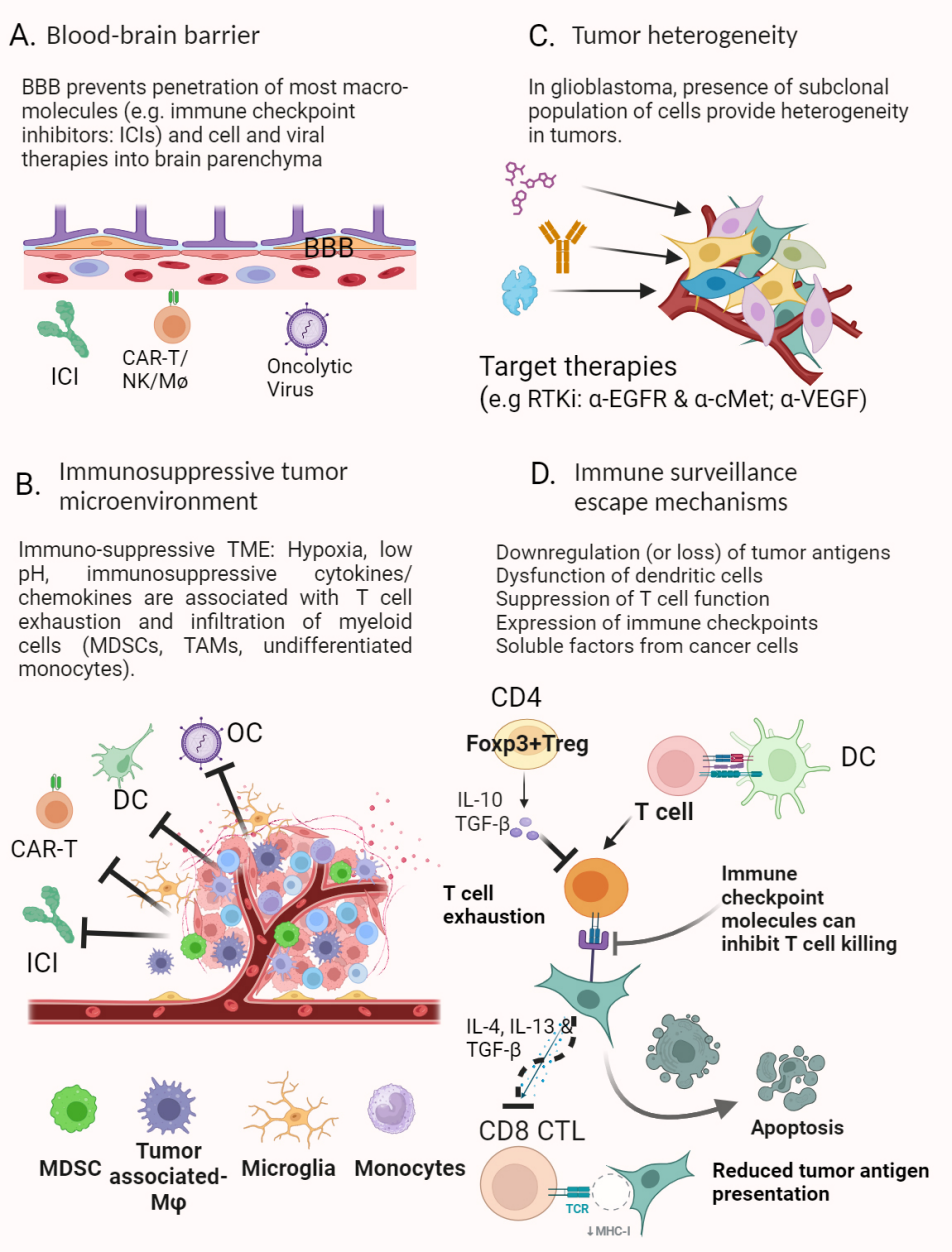
Main determinants of therapeutic failures in glioblastoma. (A) BBB can prevent the transport of most macromolecule therapeutics (e.g., immune checkpoint inhibitors), cell-based therapies, and most oncolytic viruses; (B) Within the glioblastoma, TME is a severely immunosuppressive local environment that can inhibit the function of most immunotherapies; (C) Clonal heterogeneity represents a complex problem for targeted therapeutics (e.g., receptor tyrosine kinase inhibitors and α-VEGF) to attack glioblastoma tumor cells effectively; (D) Various mechanisms for glioblastoma tumor cells to evade immune attack: tumor cells derived soluble factors (e.g., IL-4, IL-13, prostaglandin E2, and TGF-β) can suppress T cell proliferation; T cell exhaustion induced by prolonged antigen exposure can severely diminish CD8 CTL mediated cancer killing; FOXP3+ CD4 Tregs also block T cell activation. (Created with BioRender.com). BBB: Blood-brain barrier; CTL: cytotoxic T cell; DC: dendritic cell; ICIs: immune checkpoint inhibitors; MDSCs: myeloid-derived suppressor cells; TAMs: tumor-associated macrophages; TME: tumor microenvironment.

Glioblastoma tumors can produce IL-6 and drive myeloid immunosuppression by inducing PD-L1 expression on MDSCs^[85]^. Glioblastoma can also utilize the natural immune tolerance mechanisms to recruit regulatory T cells (Tregs) through the expression of indoleamine 2,3-dioxygenase (IDO)^[86]^, as well as the tumor-associated macrophages (TAMs) expression of TIM4^[87]^. Besides soluble factors, extracellular vesicles containing various signaling molecules, including growth factors, non-coding RNAs, cytokines, and other functional proteins, have been found to play an important role in the regulation of glioblastoma TME^[88]^. Those mechanisms involve an extensive network of DCs, TAMs, MDSCs, and T lymphocytes with complex and dynamic crosstalk [Figure 2].

### Heterogeneity in tumor microenvironment

Tumor heterogeneity has been well-known in glioblastoma biology at multiple levels^[89]^, including genetics/epigenetics (molecular subtypes), molecular signaling (tumor driver mutations), cellular components (clonal and subclonal tumor cells *vs.* tumor microenvironment), and temporal (primary *vs.* secondary). scRNAseq analysis of infiltrating neoplastic cells in human glioblastoma revealed vast genomic and transcriptomic heterogeneity^[90]^. Another work in brain endothelial cells derived from human glioblastoma using a similar approach (scRNAseq) showed five distinct endothelial cell phenotypes representing different states of EC activation and BBB impairment and association with different anatomical locations within and around the tumor^[91]^.

With the advancement of multi-omics platforms, tumor heterogeneity at both inter- and intra-tumoral levels has been much better depicted in glioblastoma^[92-94]^. The inter-tumoral heterogeneity can be readily appreciated by the molecular subtyping of human glioblastoma tumors by their transcriptional profile and phenotypical response to therapy^[2,95,96]^. Consistent with the four molecular subtypes of glioblastoma, a more recent scRNAseq analysis showed that glioblastoma cells can differentiate into four principal states, including astrocyte-like, oligodendrocyte progenitor-like, neural progenitor cell-like, and mesenchymal-like state^[97]^. These four cellular states are influenced by the tumor microenvironment and oncogenic drivers with certain plasticity^[97]^.

The intra-tumoral heterogeneity in glioblastoma is characterized by the presence of clonal and subclonal differentiated tumor cells, glioma stem cells (GSCs), and various components of the tumor microenvironment (stromal, endothelial, and infiltrating immune cells). A recent study by Schaettler *et al.* using scRNAseq revealed the differences between primary and secondary glioblastoma in their genomic abnormality and neoantigen formation, as well as the spatially differential T cell clones within the glioblastoma^[98]^. The authors used TCR β-chain CDR3 sequences as unique barcodes of individual T cell clones, as TCR β-chain CDR3 is highly diverse with a significant role in antigen recognition^[99]^. Their results demonstrated a topological clonal diversity of T cells in glioblastoma^[98]^. Besides microglia, another representative cell population that further complicates glioblastoma heterogeneity is a large variety of myeloid cells in the TME^[100]^. They mainly comprise TAMs, MDSCs, DCs, neutrophils, and undifferentiated monocytes^[78,101]^. Another study using scRNAseq and multiplexing tissue-imaging techniques demonstrated a spatially differential tumor microenvironment characterized by inflammatory signaling and hypoxia in glioblastoma^[102]^. The authors revealed that CD73, a critical regulator of local purinergic signaling with an essential role in inflammatory response^[103]^, was mainly expressed in glioblastoma cells with a positive correlation between levels of CD73 and HIF1α expression in the hypoxic tumor regions, where the CD73+ glioma cells co-localize with CD39+ microglia to form a spatially compartmentalized microenvironment to regulate the production of adenosine, a potent immunosuppressive metabolite^[102]^.

### Immune surveillance escape mechanisms

The crosstalk between glioblastoma and the TME through which glioblastoma tumors escape immune surveillance is very complex and highly dynamic, involving many signaling mechanisms, including both soluble factors and cell-cell interactions. Besides the BBB, which prevents drugs from reaching their target sites, these mechanisms include various immune-suppressive mechanisms, such as secretion of immunosuppressive cytokines (IL-10, TGF-β, and IL-6)^[104,105]^, expression of immune checkpoints^[106]^, and recruitment of regulatory T cells (Tregs)^[107]^, induction of M2-like phenotype of tumor-associated MΦ and microglia^[106]^, reduced tumor antigen presentation through downregulation of MHC expression, and the ability to evade immune through soluble ligands^[108,109]^ [Figure 2].

#### T cell exhaustion

T cell exhaustion is exceptionally severe in glioblastoma^[110]^, resulting in poor therapeutic efficacy of immunotherapy. Most immunotherapies focus on eliciting an anti-tumor T cell response that requires a collaboration of at least CD4 T Helper cells and CD8 cytotoxic T cells (CTLs). CD4 T Helpers can modulate antigen-specific immune response through their high plasticity and cytokine production, while CD8 CTLs exert cancer cell killing through direct cell-cell interaction and targeted release of effector molecules (perforin and granzymes)^[111]^. T cell exhaustion is mainly induced by persistent antigen exposure, and it is commonly seen in chronic infections and cancers. It is generally characterized by elevated expression of various immune checkpoints (PD-1, CTLA-4, LAG-3, and TIM-3). Remarkably, T cell exhaustion was also found to correlate with hypoxia in glioma, and both the number of exhausted T cells and the associated exhaustion markers (PD-L1, FOXO1, and PRDM1) correlated with HIF1α levels^[112]^.

The presence of regulatory T cells (Tregs) is another contributing factor for the dysregulation of T cell function in glioma TME [Figure 2]. Tregs are a subset of CD4 T cells that usually prevent autoimmunity response via suppression of inflammation and maintenance of self-tolerance^[113]^. Tregs (CD4+ Foxp3+) naturally arise from thymic differentiation^[114]^ or are induced in the already differentiated Foxp3- CD4+ T cells in the periphery^[115]^. A recent study showed that Tregs promote CD8 T cell exhaustion and restrict clonal diversity of tumor-infiltrating CD8 CTLs^[116]^. Therefore, strategies to eliminate Tregs have been developed to restore anti-tumor immunity in glioblastoma, including activation of glucocorticoid-induced tumor necrosis factor-related protein (GITR). GITR is an immune checkpoint constitutively expressed in Tregs, and its activation through ligand binding leads to the depletion of Tregs and reduced immuno-suppression. A preclinical study by Amoozgar *et al.* demonstrated that targeting Tregs with anti-GITR antibodies can relieve resistance to immunotherapy (e.g., anti-PD1) in mouse glioblastoma models^[117]^.

#### Immunosuppression by myeloid cells

A large number of myeloid cells, such as monocytes, macrophages and MDSCs, in the glioblastoma TME impose another great challenge for immunotherapy to function [Figure 2]. Among the tumor-infiltrating myeloid populations in glioblastoma, TAMs play a pivotal role in tumor progression, immunosuppression, and therapy resistance. TAMs are usually found to exhibit a tumor-promoting phenotype by producing immune suppressive cytokines such as IL-6, IL-10, and TGF-β^[104,105]^, and they represent a large population of cells with immunosuppressive function in TME. Various approaches have been proposed to target TAMs for glioblastoma treatment. For instance, by dual targeting IL-6 and CD40, Yang *et al.* showed that they could reverse TAMs-mediated tumor immunosuppression and sensitize the glioblastoma tumor to immune checkpoint inhibitors (anti-PD1 and anti-CTLA-4) in mouse tumor models^[118]^. In addition, the relatively undifferentiated monocytic MDSCs have been found to play a significant role in glioblastoma-associated immunosuppression. Domenis *et al.* demonstrate that CD14+ monocytic MDSCs were the primary mediators of the T cell suppression induced by the GSC-derived exosomes containing various immune suppressive cytokines^[119]^.

Glioblastoma can also evade immune attack by down-regulating tumor antigen expression. Tumor antigen loss during immunotherapy treatment, especially by CAR-T therapy, has been frequently reported^[120]^. Migrating or invading glioblastoma cells were found to have reduced expression of major histocompatibility complex (MHC) class I and II genes, resulting in significant down-regulation of tumor antigen presentation^[121]^. Additionally, glioblastoma TME is quite a hypoxic and acidic environment. Both hypoxia and acidosis are essential environmental cues for maintaining GSCs, especially in a HIF1α-dependent manner^[122,123]^. GSCs are believed to be primarily responsible for tumor resistance to chemotherapy and radiotherapy^[124,125]^. More importantly, GSCs have also been shown to have a significant role in the evasion of immune function^[126]^.

### Resistance to ICIs

ICIs are currently the most prevalent immunotherapy for cancer treatment. Since the approval of the first ICI (α-CTLA-4) by the FDA in 2011, these antibodies have been studied in an increasingly growing number of clinical trials, including those cancers with low response rates, such as breast cancer, cervical cancer, and brain cancer^[60,127,128]^. Despite the success of ICIs in treating hematopoietic cancers, the clinical trials in glioblastoma have been underwhelming. Besides the BBB, several contributing factors that render ICIs ineffective in glioblastoma treatment have been identified.

#### Low tumor mutational burden in glioblastoma tumor

Glioblastoma is generally considered an immunologically “cold” tumor type with a relatively lower tumor mutational burden (TMB). Thus, the neoantigen levels are also lower^[129,130]^. Higher TMB often leads to the formation of a greater number of neoantigens and a greater potential for T-cell repertoire against tumor-specific antigens^[131]^. TMB has been found to be correlated with the clinical outcome of cancer immunotherapy^[76]^. Compared with the immunologically “hot” tumor types such as melanoma and NSCLC, glioblastoma shows a much lower neoantigen burden^[132]^.

#### T cell dysfunction

Glioblastoma patients are often found to have T cell dysfunction in both CNS and peripheral blood, and T cell exhaustion is pervasive and severe in glioblastoma TME. CD8 T cell exhaustion usually starts with the loss of IL-2 production, a cytokine crucial for T cell proliferation, followed by loss or decreased production of TNF-α, IFN-γ, and granzyme B^[133]^. Tregs also make a significant contribution to the T cell dysfunction in glioma. Both natural and induced Tregs can suppress the cytotoxicity of CD8 CTLs. Tregs were found to be associated with worse prognosis in glioblastoma patients^[134]^, and it seems that the natural Tregs are the dominant subpopulation of Tregs in glioblastoma. Besides dysregulated T cell function, surprisingly, neurons have been shown to play a role in the ICI therapy resistance in glioblastoma. A recent study reported neuronal calmodulin-dependent kinase kinase-2 (CaMKK2) as a driver for the resistance to ICIs in glioblastoma^[56]^, in which CaMKK2 increased CD8 T cell exhaustion, reduced CD4 effector cell expansion, and played a role in the maintenance of immunosuppressive phenotype of tumor-associated microglia^[135]^.

#### Deficits in antigen presentation by microglia

In glioblastoma TME, antigen presentation machinery is dysregulated in almost all types of antigen-presenting cells. The immunosuppressive microenvironment in glioblastoma leads to the downregulation of MHC expression in microglia^[136,137]^. The decreased MHC expression significantly impairs the ability of microglia to effectively present antigens, limiting the activation of other immune cells and undermining the immune response against the tumor. Similarly, TAMs were found to be deficient in antigen presentation, lacking costimulatory molecules CD86, CD80, and CD40 critical for T-cell activation^[138]^. In fact, although glioblastoma tumor-infiltrating dendritic cells seemed more efficient than both MΦ and microglia in priming T-cells with exogenous antigens^[139]^, data from a preclinical study demonstrated that a better anti-tumor immunity is associated with both tumor-infiltrating dendritic cells and microglia^[140]^.

#### TAMs

A new study using patient-derived recurrent glioblastoma tumors with neoadjuvant PD-1 antibody treatment showed that α-PD-1 activated T cells and dendritic cells, but was unable to reverse the immunosuppressive phenotype in TAMs^[141]^. Work by Chen *et al.* analyzed scRNAseq data from a combined of >19,000 individual macrophages from 66 human glioma cases (50 glioblastomas and 16 low-grade gliomas) and discovered a pro-tumor subset of bone marrow-derived macrophages with the expression of a scavenger receptor MARCO^[142]^. More interestingly, this subpopulation of MARCO+ TAMs was found almost exclusively in the IDH-WT glioblastoma, and they exhibited a completely oppositive dynamic in α-PD-1 responders *vs.* non- responders^[142]^. Park *et al.* studied the immune landscape of mouse glioblastoma with α-PD-1 treatment, and found that chemokine CCL5 induced by α-PD-1 treatment seemed to recruit the anti-inflammatory TAMs into the glioblastoma TME^[143]^. A CyToF-based high-plexing immune profiling approach revealed that ICI-sensitivity in both human and mouse tumors was associated with a higher number of T cells and dendritic cells (DCs) and a lower number of PD-L1 positive TAMs^[144]^.

#### Anti-inflammatory glucocorticoids

Glucocorticoids have been used to control certain adverse effects associated with cancer immunotherapy. Interestingly, concurrent administration of dexamethasone, a potent corticosteroid frequently used in glioblastoma patients to decrease tumor-associated edema, has been shown to be detrimental to immunotherapy for patients with glioblastoma^[145]^. Though the clinical data in this study was limited to a subset of patients with wild-type IDH-1 glioblastoma under α-PD-L1 treatment, the concurrent dexamethasone diminished the response to α-PD-1 therapy in two different mouse glioma models^[145]^. It is worth mentioning that glioblastoma patients under standard (radiation plus TMZ) treatment who received dexamethasone treatment also showed a worse outcome^[146]^. However, this is likely because MGMT promoter contains two nonconsensus glucocorticoid-responsive elements and glucocorticoids can upregulate MGMT expression^[147]^. A comprehensive study of MGMT promoter activity in glioblastoma cell lines further clarified that dexamethasone, but not TMZ or irradiation, can induce the upregulation of MGMT expression via a SP-1 dependent fashion^[148]^, while not through altering the epigenetic status (i.e., methylation) of the MGMT promoter.

### Role of non-coding RNAs

Long non-coding RNAs (LncRNAs) have been increasingly recognized for their essential role in cell growth, survival, proliferation, pluripotency, and immune functions correlating to the malignant transformation of normal cells into cancerous cells^[149-151]^. MALAT1, NEAT1, and H19 are among the common LncRNAs that influence the response of glioblastoma/glioma to chemotherapeutics^[152]^. Another lncRNA, LINC00021, was significantly upregulated in glioblastoma, especially in the TMZ resistance cells or tissues, enhancing resistance to TMZ through Notch pathway and epigenetically silencing p21 expression^[48]^. A study also showed that LncRNA SNHG15 promotes pro-glioblastoma cytokines TGF-β and lL-6 in TMZ-resistance cells via M2-polarization of microglial cells^[153]^.

Micro RNA (miRNA) also plays a role in the regulation of glioblastoma TME. One example is the miR-15/16 cluster, which was found to be differentially expressed in various human cancers such as glioma and prostate cancer^[154,155]^. In a mouse glioblastoma model, Yang *et al.* demonstrated that loss of miR-15/16 in mice carrying GL261 tumors resulted in improved survival, enhanced CD8 T cell infiltration, and reduced expression of T cell exhaustion markers (PD1, TIM-3, and LAG-3)^[156]^. An *in vitro* study by Hubner *et al.* identified miR-93 as an anti-inflammatory tumor suppressor in glioblastoma^[157]^. Their data showed that miR-93 was downregulated in human glioblastoma cell lines, and restoration of miR-93 levels in glioblastoma cells led to a decreased expression of an array of inflammatory genes (HIF-1α, MAP3K2, IL-6, G-CSF, IL-8, LIF, and IL-1β)^[157]^. More interestingly, TCGA data mining confirmed that high expression of miR-93 was associated with better survival in the MGMT-methylated cohort of glioblastoma patients.

## OPPORTUNITIES

### Approaches to alter immuno-suppression in glioblastoma TME

Many great efforts have been made to overcome the difficulty of immunotherapy applications in neuro-oncology. For example, a clinical trial found that neoadjuvant PD-1 blockade resulted in significantly improved overall survival and progression-free survival in patients with recurrent glioblastoma^[158]^. In this study, patients received anti-PD1 treatment ~2 weeks before surgery, and the PD1 antibody was able to elicit both systemic and local anti-tumor immunity. Other attempts are primarily focused on modulating the immune suppression in the glioblastoma tumor microenvironment by targeting various components of the TME, such as TAMs and MDSCs (summarized in a recent review by Wang *et al.*^[159]^). In the meantime, new targets have been identified for future immunotherapy development. For instance, TAMs associated CD73 was found to be a promising target with potentially synergistic effects along with dual inhibition of PD1 and CTLA-4^[160]^. CD47/SIRPα axis is another exciting target to consider. SIRPα governs the phagocytosis activity of MΦ. When CD47 on the cancer cell surface engages with SIRPα on MΦ, it sends a “Don’t-eat-me” signal to prevent phagocytosis of cancer cells by MΦ. Treatment with anti-CD47 plus TMZ was shown to activate both innate and adaptive anti-tumor immunity in a preclinical study^[161]^.

A single-cell RNA-seq study of patient glioma infiltrating T cells revealed CD161 (KLRB1) as a promising immunotherapy target. Depleting CD161 led to T cell activation and anti-tumor immunity both *in vitro* and *in vivo*^[162]^. An independent study using data from a large cohort of glioma patients confirmed that CD161 might play an important role in promoting glioma progression via inhibition of T cell function^[163]^.

Besides checkpoint inhibition, a deeper understanding of the resistance mechanism to CAR-T therapy in solid tumors was achieved through a genome-wide CRISPR knockout screen in glioblastoma^[164]^. A recent study using a genome-wide CRISPR knockout screen in glioblastoma revealed a functional requirement of IFN-γ receptor in glioblastoma for sufficient adhesion of CAR-T cells to mediate productive cytotoxicity^[164]^. This study suggests that strategies to enhance the binding of CAR-T cells to the solid tumor will likely result in a better treatment response. Another strategy to enhance the infiltration of CAR-T cells into glioblastoma tumors by combining CAR-T with a CXCL11-armed oncolytic virus also demonstrated an improved anti-tumor immunity in a syngeneic mouse glioma model^[165]^.

### Combinatorial approaches and new forms of immunotherapies

Combination therapy has been extensively explored to improve glioblastoma treatment. For instance, resistance to α-VEGF monotherapy was common in glioblastoma. A new study reported that combined blockade of VEGF, Angiopoietin-2, and PD1 could reprogram glioblastoma endothelial cells into quasi-antigen-presenting cells and induced a durable anti-tumor T cell response^[166]^. A recent review has nicely summarized the current status of combinatorial approaches, including both chemo- and immunotherapies, for glioblastoma treatment^[167]^. Additionally, many new forms of immunotherapy are emerging with great hope to shift the paradigm of glioblastoma treatment. A recent study reported a nanoporter (NP)-hydrogel complex for local induction of CAR-macrophages (CAR-MΦ) targeting CD133+ glioblastoma stem cells in tumor resection cavity with promising results^[168]^. This nanomicelle complex consists of a self-assembled peptide-based hydrogel loaded with the CD133-targeting CAR construct and then was coated with a citraconic anhydride–modified dextran with the ability to bind to CD206, a typical surface marker of M2 macrophages. Different from the *ex vivo* engineering of CAR-MΦ developed by Klichinsky *et al.*^[169]^, the nanoporter-hydrogel-based in situ induction of strategy CAR-MΦ largely simplified the process of CAR-MΦ preparation and minimized potential systemic toxicity from CAR-MΦ.

The CAR-NK cells have also been explored to treat glioblastoma either by Her2 targeting monotherapy^[170]^ or in combination therapy. For instance, the Off-the-Shelf EGFR-targeting CAR-NK cells have been tested in combination with an oncolytic virus expressing the IL15/IL15Ralpha complex and the combinatorial therapy demonstrates a strong anti-tumor immunity^[171]^. A significant problem associated with CAR-NK cell therapy is the shedding or down-regulation of the ligands in cancer cells that bind natural killer group 2D (NKG2D) receptors on the natural killer (NK) cells. NKG2D is an activating receptor widely expressed in NK cells as well as in some subsets of T cells^[172]^. To overcome the limitation of NKG2DL heterogeneity in the tumor, a recent study using a bispecific antibody with two ScFv fragments (linked with a IgG4-Fc) that target Her2 (tumor) and NKG2D (NK cells), respectively, in combination with human NK-92 cells, showed synergistic tumor cell killing effects in both *in vitro* and *in vivo* conditions^[173]^. Although the syngeneic tumor model they used represents a situation of a heterogenous expression of NKG2DLs in tumor cells, the flank tumors they used did not address the difficulty in delivery of the combination therapy across the BBB^[173]^.

Another interesting phenomenon is the sex difference in response to immunotherapy in glioma. The sex disparity in brain cancer has been reported by several groups^[174-177]^. A recent meta-analysis revealed that female patients with glioblastoma treated with immunotherapy had a statistically significant survival advantage in overall survival over their male counterparts^[178]^. They also found that female patients exhibited a more robust survival advantage with cancer vaccine treatment. Another study by Bayik *et al.* discovered that two subsets of myeloid-derived suppressor cells (MDSCs) have a sex-specific tumor-promoting phenotype in both mouse and human glioblastoma^[179]^. All these data suggest that a more personalized approach, which at least considers sex differences in glioblastoma treatment, will more accurately evaluate the efficacy of immunotherapy.

### New drug delivery technologies to overcome BBB limitation and activate glioblastoma TME

**Figure 3:**
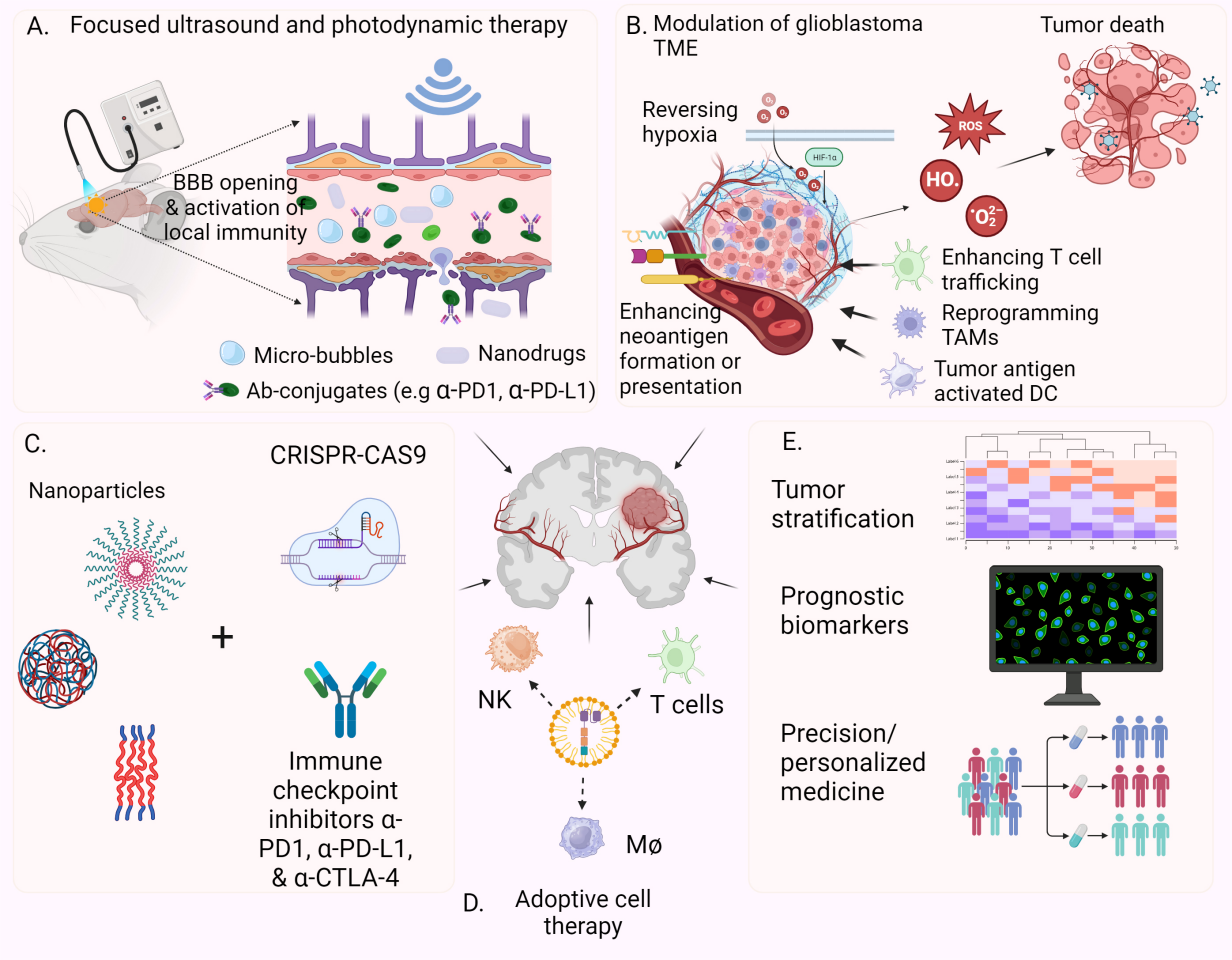
Potential new approaches to improve glioblastoma treatment. (A) The focused ultrasound in combination with micro-bubbles and photodynamic therapy (PDT) can temporarily open BBB to allow therapeutics crossing. PDT can also activate local immunity in TME; (B) New approaches to modulate glioblastoma TME by targeting hypoxia, activating suppressed local immunity, or enhancing cancer neoantigen formation in tumor cells; (C) Novel nanodrug delivery technologies in combination with CRISPR/Cas9-based gene editing and immune checkpoint inhibitors; (D) Various forms of adoptive cell therapies; (E) Better strategies for tumor stratification, prognostic prediction and personalized medicine would enhance the clinical outcome of glioblastoma treatment. (Created with BioRender.com). BBB: Blood-brain barrier; DC: dendritic cell; TAMs: tumor-associated macrophages; TME: tumor microenvironment.

Various new technologies have demonstrated promising progress in overcoming BBB, and we summarized a few new approaches with great potential to improve the glioblastoma treatment outcome [Figure 3]. Among those new approaches, the use of ultrasound to open BBB for glioblastoma treatment has been applied in several areas, including immunotherapy delivery. Using low-intensity pulsed ultrasound to temporarily disrupt BBB, Sabbagh *et al.* demonstrated a significantly improved BBB penetration of both anti-PD1 antibody and EGFRvIII targeting CAR-T cells, as well as significantly improved survival in mouse glioblastoma models^[180]^. Another study by Sheybani *et al.* applied MRI-guided focused ultrasound with systemic injection of microbubbles and studied the impact of this approach on temporary BBB disruption in a mouse glioma model^[181]^. This approach caused a transient local inflammatory phenotype in the mouse glioblastoma, with an increased number of dendritic cells and the upregulated maturation marker. However, they did not see a significant increase in CD8 T cells in the TME^[181]^.

Another technology to modulate BBB function is photodynamic therapy (PDT). Conventionally, PDT relies on a photosensitizer, such as 5-aminolevulinic acid (5-ALA)^[182]^, that can accumulate in tumor tissue, plus a laser that can stimulate the photosensitizer, followed by energy transfer to generate reactive oxygen species, leading to damages to the cancer cells^[183]^. It is noteworthy that PDT has shown promise in temporary opening of BBB, possibly through modulating certain components of TJs^[184]^. Interestingly, PDT can also induce an acute inflammatory response in which both innate and adaptive immune systems are activated^[185]^. Recently, BBB opening was shown to affect the meningeal lymphatic system characterized by an anti-tumor effect of talaporfin sodium (TS)-PDT as well as its synergy with the immune checkpoint inhibitor^[186]^. *In vitro* studies have demonstrated that targeted TS-PDT triggers various forms of cell death, including apoptosis, necrosis, and autophagy-associated cell death. Furthermore, TS-PDT induces the acute activation of lymphatic drainage in the brain and the clearance of unwanted molecules from the CNS^[187,188]^. The approval of 5-ALA by the FDA for fluorescence-guided glioblastoma resection has sparked a renewed interest in its potential application for PDT^[182]^.

Nanotechnology has also made significant advancements in the field of glioblastoma treatment. Various forms of nanomedicines have exploited the features of the glioblastoma tumor microenvironment for efficient BBB crossing and release of payloads^[189-191]^. Fan *et al.* engineered an MMP-2-activated nanoparticle to carry anti-CD276 & CD3 bispecific antibodies and demonstrated that this strategy enhanced IFN-γ-induced tumor cell ferroptosis^[192]^. A polylactic-co-glycolic acid (PLGA) nanoparticle encapsulated disulfiram was used to block hypoxia-induced NF-κB signaling and glioma stem cells^[193]^. Zou *et al.* devised a polymer-based CRISPR-Cas9 nano-capsule for systemic gene therapy delivery to glioblastoma^[194]^. This nano-capsule has both the BBB crossing and tumor targeting functions mediated through an angiopep-2 peptide^[195]^. By targeting polo-like kinase (PLK-1) via a sgRNA, the strategy demonstrated a significant survival advantage over the control mice^[194]^.

## CONCLUSION

Despite advances in surgical technologies and therapeutics development, there has been limited improvement in the long-term survival rate of glioblastoma patients, with a 5-year survival still around 5%-10%. Many lessons have been learned in glioblastoma drug resistance mechanisms, especially with cutting-edge scRNAseq, spatial biology, and other-omics platforms. Efforts are needed to overcome BBB and tumor heterogeneity, targeting glioma stem cells and their niches, enhancing T cell trafficking and preventing their exhaustion, and modulating the immunosuppressive TME in glioblastoma. A complex disease, such as glioblastoma, would require a complex solution. Multidisciplinary approaches involving nanodrug carriers, focused ultrasound, plus temporary BBB permeability enhancement technologies (micro-bubbles, phototherapy) in combination with gene and immuno-therapy will likely lead to an improved outcome [Figure 3]. In addition, a much less traveled path is to enhance glioblastoma neoantigen formation. Glioblastoma tumors have a relatively lower TMB, which was shown to correlate with immunotherapy outcomes in solid tumors^[76,196]^. Lower TMB results in lower neoantigen generation, which enables a stealth mode of glioblastoma cells. Therefore, increasing the formation of neoantigens may significantly promote tumor recognition and clearance by the immune system^[197]^. Besides T cells, strategies to activate other infiltrating immune cells (TAMs, microglia, and MDSCs) that reside in the glioma TME in large abundance may effectively reverse the local immunosuppression. Finally, a more precise tumor stratification approach and improved prognostic biomarkers will help determine the most effective combinatorial therapies for glioblastoma treatment.

## DECLARATIONS

### Copyright

© The Author(s) 2023.
